# A Case of Systemic Chemotherapy With Paclitaxel/Cisplatin Followed by Wide Local Vulvectomy in Pelvic Lymph Nodes-Related Stage IVB Vulvar Cancer

**DOI:** 10.7759/cureus.60432

**Published:** 2024-05-16

**Authors:** Junsuke Muraoka, Naotake Tanaka, Takahiro Sugiyama, Makiko Itami, Kiyomi Suzuka

**Affiliations:** 1 Department of Gynecology, Chiba Cancer Center, Chiba, JPN; 2 Department of Surgical Pathology, Chiba Cancer Center, Chiba, JPN

**Keywords:** vulvar neoplasm, surgery, female, drug therapy, cancer

## Abstract

Multimodality treatments, including chemotherapy, radiation, and surgery, have been evaluated to reduce the extent of resection and morbidity in patients with advanced vulvar cancer. Here, we report the case of a 55-year-old woman diagnosed with advanced vulvar cancer with inguinal and pelvic lymph node metastasis. She exhibited cancerous labia, which were entirely covered with ulcerated and exophytic lesions of squamous cell carcinoma, and underwent systemic chemotherapy consisting of combined paclitaxel-cisplatin. After eight cycles of this regimen, the tumors had nearly regressed, and we performed a wide local vulvectomy with a plastic musculocutaneous flap. Pathological examination revealed no residual carcinoma in the excised labia, indicating that the chemotherapy elicited a pathological complete response. The paclitaxel-cisplatin regimen may provide sufficient efficacy for selected patients with stage IVB vulvar cancer. In addition, surgical strategies should be tailored to avoid complications associated with extensive surgery and more emphasis should be placed on the patient’s expected quality of life.

## Introduction

Vulvar cancer is a rare disease that accounts for 4% of all gynecological malignancies and occurs in 0.85/100,000 women per year worldwide (approximately 44,000 total new cases were estimated in 2018) [1,2]. The 1-year and 5-year survival rates of advanced-stage IVB vulvar cancer were estimated at 44.7% and 18.3%, respectively [3]. Cancer remains a disease of older adults; however, recent studies indicate that the incidence is rising in younger women, most likely because of exposure to human papillomavirus (HPV), whereas older patients tend to be HPV-negative [2].

Treatment recommendations for vulvar cancer have been published in several guidelines, including the International Federation of Gynecology and Obstetrics (FIGO) staging system [3] and the Japan Society of Gynecologic Oncology [4]. Treatment for vulvar cancer typically includes resection, radiation, chemotherapy, and palliative therapy, which varies by disease stage. However, considering the rarity of the disease, performing prospective and randomized clinical trials is challenging. Thus, obtaining high-quality data to determine the preferred chemotherapeutic drug regimen for patients with advanced-stage cancer is difficult. Moreover, current surgical strategies have evolved the paradigm to promote less extensive excision as opposed to radical vulvectomy [5].

Here, we described a successful case of stage IVB vulvar cancer with pelvic node metastasis treated with combined paclitaxel-cisplatin (TP) chemotherapy followed by wide local vulvectomy, which resulted in a favorable response.

## Case presentation

**Figure 1:**
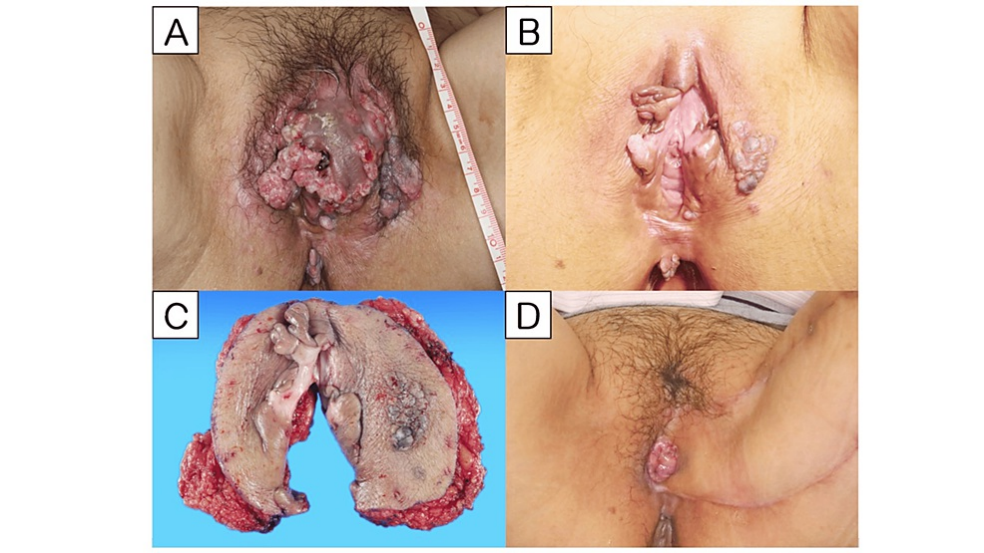
Macroscopic appearance of vulval lesions 1A shows the initial finding of vulval lesions, which regressed after six cycles of systemic TP chemotherapy as shown in 1B. The excised specimen at the wide local vulvectomy and the wound appearance two months after surgery are presented in 1C and 1D, respectively.

**Figure 2:**
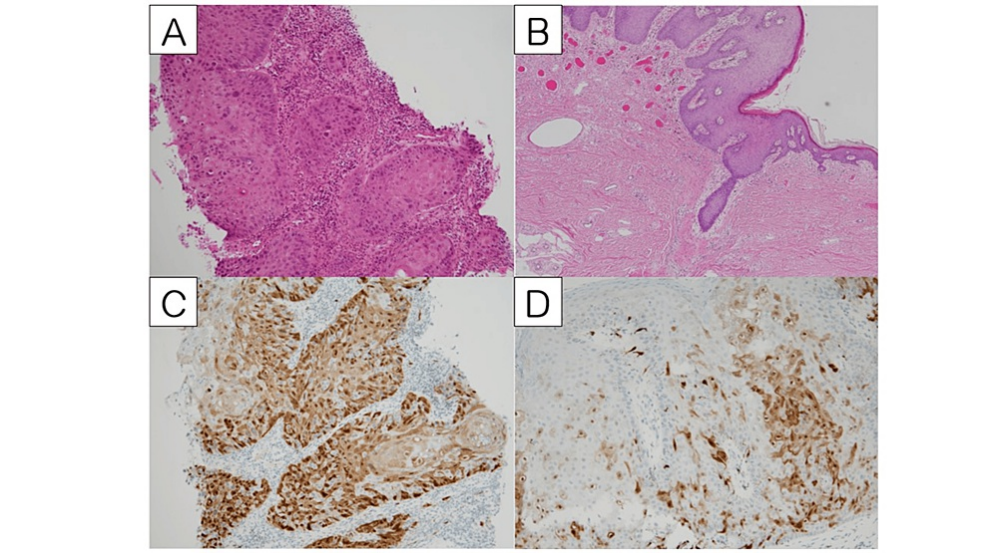
Histopathology findings of vulval lesions Histopathology findings in H&E and p16 immunostaining sections. 2A shows invasive SCC of the initial cancerous lesion of the labia, and 2B shows the excised specimen, which is composed only of VIN3 after a total of eight cycles of systemic TP chemotherapy. As shown in 2C and 2D, both the invasive SCC and VIN specimens show non-block-type p16 immunostaining, indicating HPV-independent lesions. SCC: squamous cell carcinoma; VIN: vulvar intraepithelial neoplasia

**Figure 3:**
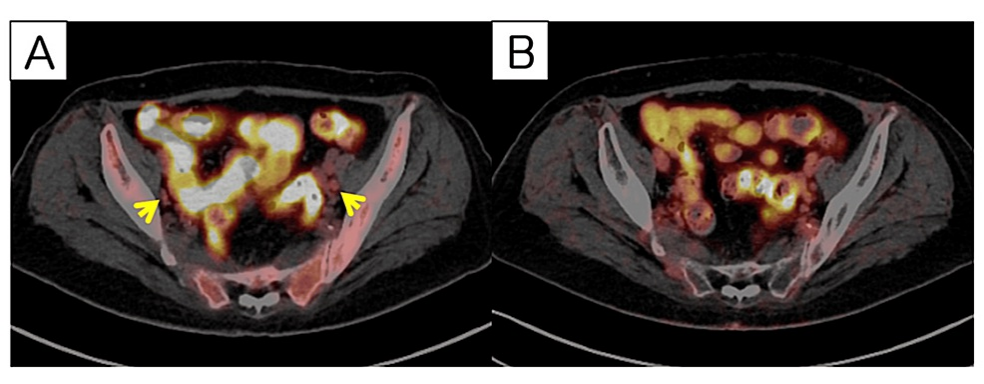
FDG-PET/CT images at initial diagnosis and after systemic TP chemotherapy FDG-PET/CT images at initial diagnosis are shown in 3A and after systemic TP chemotherapy are shown in 3B. The yellow-colored arrowheads indicate metastatic pelvic lymph nodes with moderate accumulations of FDGs, and these abnormal accumulations disappeared after chemotherapy.

A 55-year-old Japanese woman, gravida 5 para 3, presented with a vulvar mass. The patient also had type 2 diabetes mellitus with a hemoglobin A1c level of 10.3%, which required insulin therapy. In addition, she was given medication for condyloma acuminatum at 40 years of age but left it untreated. Physical examination initially revealed that her labia were covered entirely with ulcerated and exophytic lesions spread closely to the meatus urethra (Figure 1A). Bilateral inguinal lymph nodes were clinically palpable. A biopsy revealed invasive squamous cell carcinoma of the vulva with non-block-type p16 immunostaining (Figure 2A and Figure 2C). 18F-fluorodeoxyglucose positron emission tomography and computed tomography (FDG-PET/CT) imaging showed a high level of FDG accumulation (SUVmax ≤10.90) in the bilateral inguinal lymph nodes and a moderate level of FDG accumulation (SUVmax ≤7.17) of lymph nodes in the bilateral obturator and iliac regions (Figure 3A), which confirmed a diagnosis of International Federation of Gynecology and Obstetrics (FIGO) stage IVB vulvar cancer. No tumor involvement was observed beyond the pelvis, including the lung, liver, or elsewhere. Considering her satisfactory performance status (PS), informed consent for chemotherapy was provided by the patient, and systemic chemotherapy consisting of 175 mg/m^2^ of paclitaxel and 50 mg/m^2^ of cisplatin once every three weeks was initiated.

The vulvar mass showed marked regression with progressive chemotherapy (Figure 1B). Biopsy from her labia after six cycles of chemotherapy revealed residual malignant cells on the right side but only vulvar intraepithelial neoplasia (VIN) 1 on the left side. FDG-PET/CT imaging revealed no FDG accumulation among the intrapelvic or inguinal lymph nodes (Figure 3B). The patient experienced grades 1-2 of chemotherapy-induced peripheral neuropathy according to the Common Terminology Criteria for Adverse Events (CTCAE) version 5.0, and she took pregabalin, but her PS was 1. Consequently, we performed palliative surgery for the remaining vulvar cancer. She withheld assent to perform lymphadenectomy for fear of developing lymphedema. After two additional cycles of TP chemotherapy, the patient underwent a wide local vulvectomy with a plastic musculocutaneous flap (Figure 1C). The surgical procedure was performed as follows: the tumors were removed 2 cm beyond the edge of the tumorous lesion, and the meatus urethra and anus were preserved. The gracilis muscle flap was used to reconstruct the left side of the vulva. Two weeks after surgery, the right side, nonplastically built side of the wound opened; thus, we continued to irrigate the dehiscence. Two months after surgery, the wound was well-adapted and the patient was able to urinate and defecate normally (Figure 1D).

Examination of the resected labia showed VIN with non-block-type p16 immunostaining (Figure 2B and Figure 2D) and revealed no remaining viable malignant cells and adequate surgical margin. The patient underwent physical examination and laboratory testing once in three months. In addition, chest/abdomen/pelvis CT imaging and annual cervical/vaginal cytology tests were conducted once in six months to evaluate nodal status and recurrence, but no cancer relapse occurred one year after surgery without adjuvant therapy.

## Discussion

We report a successful case of a patient with stage IVB vulvar cancer who underwent TP chemotherapy followed by a wide local vulvectomy, which resulted in a favorable response. Systemic chemotherapy is an effective therapeutic strategy for shrinking cancer and avoiding complications associated with aggressive surgery for stage IVB vulvar cancer with pelvic lymph node metastasis. Less extensive surgery for patients with advanced-stage disease can improve organ preservation and maintain their quality of life (QOL).

Typically, the concurrent use of external beam radiotherapy with chemotherapy offers a chance of cure in women with advanced-stage or large-sized tumors [6]; however, it is often associated with high morbidity (e.g., fibrosis, stenosis, and vulvovaginal atrophy). Compared with concurrent chemoradiation, systemic chemotherapy offers the advantages of local morbidity reduction and treatment of occult or distant diseases. In previous studies by Wagenaar et al. [7] and Aragona et al. [8], systemic chemotherapy resulted in >50% response rates and increased surgical feasibility for initially inoperable patients. These studies used combined bleomycin, methotrexate, and lomustine or cisplatin-based chemotherapy; however, currently, the National Comprehensive Cancer Network recommends TP chemotherapy for advanced or metastatic vulvar cancer [9]. Moreover, Raspagliesi et al. reported that six women with stage III/IV vulvar cancer who received TP chemotherapy experienced a 66% response rate [10]. Our present case experienced a good clinical response after six cycles of TP chemotherapy and surgical resection. After a total of eight cycles of chemotherapy, a favorable response without severe chemotherapy-related toxicity was achieved. Recent studies have evaluated the addition of bevacizumab as an adjunctive chemotherapy. Furthermore, a phase 2 study of pembrolizumab, an anti-programmed cell death 1 monoclonal antibody, resulted in an objective response rate of 10.9% in patients with advanced vulvar cancer [11].

We believe that the final diagnosis is established by histological examination of lymph node specimens as well as the primary tumor. When metastatic involvement of lymph nodes and/or advanced disease is suspected, whole-body CT with intravenous contrast or FDG-PET/CT should be performed to exclude pelvic lymph node involvement and the presence of other distant metastases. Several guidelines recommend that the suspicious inguinal nodes should be assessed by ultrasound-guided fine-needle aspiration or core needle biopsy if this would alter the primary treatment. In addition, equivocal distant metastasis should be biopsied, whenever possible, to analyze the suspicious metastatic lesions. In the present case, we made a diagnosis of inguinal and pelvic lymph node metastasis because of the physical examinations of her inguinal mass and the findings of moderate to high-level accumulations of FDGs, although we appreciated the significance of lymph node sampling for staging. Unfortunately, the attempt to identify the metastatic pelvic lymph nodes by CT-guided biopsy was abandoned because it would be invasive and technically challenging due to the localization of the lymph nodes.

In the present case, the metastatic lymph nodes had completely regressed following TP chemotherapy, whereas residual FDG accumulation was located exclusively within the labium. Therefore, we performed wide local excision for the remaining vulvar cancer. Because the scarring lesion of the labium was located laterally wide on the left side, the wound was reconstructed with a left gracilis muscle flap by the plastic surgeons. The gracilis myocutaneous flap is useful in cases of largely denuded defects in the perineum resulting from radical vulvectomy or perineal surgery, in which primary closure may result in postoperative dehiscence of the wound incision [12]. Our case decided not to receive lymphadenectomy, deeply concerned about the development of lymphedema. A Japanese nationwide survey investigated that inguinofemoral lymphadenectomy was performed in approximately 60% of cases among initially operable patients with vulvar cancer [4]; however, it was found to be associated with serious adverse effects, so the risks and benefits of the intervention need to be fully explained to patients. Because chemotherapy can effectively shrink the tumor and secure a sufficient margin to remove the tumor apart from the urethra and anus, tailored surgical strategies for preservation of urination and defecation can be administered. For patients with early vulvar cancer, individualized surgical treatments are reported to be safe and effective [13]; however, insufficient results for the long-term prognosis may occur when performing less extensive surgery after systemic chemotherapy for locally advanced vulvar cancer. Therefore, long-term surveillance is needed.

The issue that needs to be discussed is whether radiation should be offered in this case or not. Radiation to the pelvis is commonly administered with a primary or adjuvant intent for many gynecological cancers. For patients with stage IVB vulvar cancer presenting with unfavorable conditions, palliative radiotherapy would be considered as an option for primary treatment; however, we suggested systemic chemotherapy for the initial treatment, considering her satisfactory PS. In addition, pathological examination of wide local vulvectomy revealed no malignant cells remaining in the excised specimen and therefore we conducted surveillance without performing adjuvant radiotherapy.

## Conclusions

Our present case was diagnosed with advanced vulvar cancer with inguinal and pelvic lymph node metastasis. The patient underwent TP chemotherapy followed by surgery, which resulted in a favorable response. Systemic chemotherapy consisting of a TP regimen for locally advanced vulvar cancer is beneficial for tumor shrinkage for selected patients with stage IVB vulvar cancer. Physicians should individualize surgical strategies for improving organ preservation and reducing complications associated with extensive surgery and emphasize the patient’s QOL.
